# Assessing the Relationship Between Race, Language, and Surgical Admissions in the Emergency Department

**DOI:** 10.5811/westjem.2022.10.57276

**Published:** 2023-02-01

**Authors:** Grant H. Rigney, Soham Ghoshal, Sarah Mercaldo, Debby Cheng, Jonathan J. Parks, George C. Velmahos, Michael H. Lev, Ali S. Raja, Efren J. Flores, Marc D. Succi

**Affiliations:** * Harvard Medical School, Boston, Massachusetts; † Massachusetts General Hospital, Department of Radiology, Boston, Massachusetts; ‡ Massachusetts General Hospital, Medically Engineered Solutions in Healthcare Incubator, Innovation in Operations Research Center (MESH IO), Boston, Massachusetts; § Massachusetts General Hospital, Department of Surgery, Boston, Massachusetts; || Massachusetts General Hospital, Department of Emergency Medicine, Boston, Massachusetts

## Abstract

**Introduction:**

English proficiency and race are both independently known to affect surgical access and quality, but relatively little is known about the impact of race and limited English proficiency (LEP) on admission for emergency surgery from the emergency department (ED). Our objective was to examine the influence of race and English proficiency on admission for emergency surgery from the ED.

**Methods:**

We conducted a retrospective observational cohort study from January 1–December 31, 2019 at a large, quaternary-care urban, academic medical center with a 66-bed ED Level I trauma and burn center. We included ED patients of all self-reported races reporting a preferred language other than English and requiring an interpreter or declaring English as their preferred language (control group). A multivariable logistic regression was fit to assess the association of LEP status, race, age, gender, method of arrival to the ED, insurance status, and the interaction between LEP status and race with admission for surgery from the ED.

**Results:**

A total of 85,899 patients (48.1% female) were included in this analysis, of whom 3,179 (3.7%) were admitted for emergent surgery. Regardless of LEP status, patients identifying as Black (odds ratio [OR] 0.456, 95% CI 0.388–0.533; P<0.005), Asian [OR 0.759, 95% CI 0.612–0.929]; P=0.009), or female [OR 0.926, 95% CI 0.862–0.996]; P=0.04) had significantly lower odds for admission for surgery from the ED compared to White patients. Compared to individuals on Medicare, those with private insurance [OR 1.25, 95% CI 1.13–1.39; P <0.005) were significantly more likely to be admitted for emergent surgery, whereas those without insurance [OR 0.581, 95% CI 0.323–0.958; P=0.05) were significantly less likely to be admitted for emergent surgery. There was no significant difference in odds of admission for surgery between LEP vs non-LEP patients.

**Conclusion:**

Individuals without health insurance and those identifying as female, Black, or Asian had significantly lower odds of admission for surgery from the ED compared to those with health insurance, males, and those self-identifying as White, respectively. Future studies should assess the reasons underpinning this finding to elucidate impact on patient outcomes.

## INTRODUCTION

### Background

Racial inequities harm the health of racially and ethnically marginalized individuals.^1^ Racism has been shown to adversely affect nearly all facets of the healthcare system, from insurance status^2^ to clinician ratings of pain levels^3^ to readmission after surgery.^4^ Even when controlling for variables known to influence health outcomes such as insurance status, education, and income, the effects of racism on health remain significant and play independent and likely causal roles in health disparities.^5^

Approximately 67.3 million people in the United States speak a language other than English at home,^6^ and recent estimates suggest that 1 in 10 working-age Americans have limited English proficiency (LEP), a term used to describe not being fluent in the English language. Limited English proficiency individuals are known to have poorer quality and less access to healthcare when compared to those with English proficiency.^7^ Previous studies have shown that patients with LEP experience increased postoperative hospital admissions, significantly increased risk of infection,^8^ and more unplanned ED revisits.^9^ Further, patients with LEP have also been shown to have increased in-hospital mortality rates, as well as an increased frequency of major adverse cardiovascular and cerebrovascular events.^10^ The combined effects of race and English language proficiency have been relatively understudied, in part due to many studies combining language and race variables, thereby preventing any independent measurement. Current literature suggests that LEP and race are related and, although they serve as potential confounders, impact different aspects of a patient’s health journey.

While sociocultural factors such as race, English proficiency, and ethnicity are understood to impact ED and inpatient quality of care, the need to undergo emergency surgery is not often clearly or directly influenced by such factors. Thus, examining urgent surgery procedures provides a unique opportunity to evaluate the impact of racism and language proficiency on surgical care delivery.

### Objectives

Our goal was to assess the impact of racism – with race and LEP status as proxy measures thereof – on rates of admission for emergent surgery from the ED.

## METHODS

### Study Design and Setting

We conducted this single-hospital, retrospective observational study at a 1,011-bed quaternary care, urban, academic medical center treating approximately 110,000 ED patients annually. With 66 beds, the ED serves as a Level I trauma center, a Level I burn center, and a comprehensive stroke and ST-elevation myocardial infarction center.^11^–^13^ The study was compliant with the Health Insurance Portability and Accountability Act and was approved with exemption by the study site’s institutional review board.

### Participants

To exclude major volume changes in the ED due to the coronavirus 2019 pandemic, we analyzed all patients (adult and pediatric) presenting to the ED between January 1–December 31, 2019 who were also admitted for surgery from the ED. Importantly, we only included patients who had surgery and did not include patients who were admitted for a surgical indication (eg, small bowel obstruction) but did not ultimately undergo surgery. Surgery was defined via indication in the electronic health record (EHR) and included minor (eg, drain placement) surgical procedures, although these were a minority (<5%) of the surgical cases. Participants were identified using the EHR (Epic Systems, Verona, WA).^14^ We extracted records for all ED admissions and all surgeries performed during the study period.^15^,^16^ This data was then cross-referenced to identify individuals who presented to the ED and underwent surgery on the day of or day after admission to the ED. Undergoing surgery the day of or the day after admission was defined as “emergent” surgery in this study.

**Population Health Research Capsule**

*What do we already know about this issue?*

*Racism adversely affects many facets of the healthcare system, including patient insurance status, clinician ratings of pain levels, and readmission after surgery*.

*What was the research question?*

*What is the impact of race and limited English proficiency on admission for emergency surgery from the emergency department?*

*What was the major finding of the study?*

*Compared to White patients, those identifying as Black (OR 0.456, 95% CI 0.388–0.533; P<0.005) or Asian (OR 0.759, 95% CI 0.612–0.929; P=0.009) had significantly lower odds for admission for surgery. Females similarly had lower odds for admission than males (OR 0.926, 95% CI 0.862–0.996]; P=0.04), but we found no difference in English language proficiency*.

*How does this improve population health?*

*Our data contributes to research evaluating the impact of widespread surgical disparities experienced by Black, Asian, and female patients*.

Participants were excluded if they were missing data on the use of an interpreter, preferred primary language, method of arrival to the ED (eg, public transportation, car, ambulance), or if they were dead on arrival at the ED. We did not exclude any patients based on criteria of frequent ED use, as we sought to maximize our detection of patients who were admitted for surgery. We placed no restrictions on the type of surgery for which patients were admitted. Patients were considered to be LEP if they used hospital interpreter services. Of note, patients were excluded if their method of arrival to the ED was unavailable because we believed it would be a significant confounder of their likelihood of being admitted for emergent surgery if not controlled for (ie, arriving via medical flight is associated with more severe illness than via public transportation and thus increases the chances the patient will be admitted for surgery). We analyzed only individuals with LEP who used hospital interpreter services.

### Outcome Measures and Data Collection

The primary outcome for this study was direct admission for surgery from the ED. For each patient, we obtained the following data: age; gender; race; ethnicity; whether they were admitted for surgery; whether they used a hospital interpreter; insurance status; and their method of arrival to the ED. Patients who self-reported they were of Hispanic ethnicity were automatically considered to be of Hispanic race; however, all other self-reported races were taken from the race category in the EHR instead of the ethnicity column. This was required due to an error in the EHR data retrieval system that did not report race for individuals who selected Hispanic in the chart.

### Statistical Methods

We compared the distribution of demographic variables by those patients admitted for surgery from the ED and those patients not admitted for surgery using the Wilcoxon test (for continuous variables) and Pearson’s chi-square test (for categorical variables). A multivariable logistic regression model was fit to examine the odds of admission for surgery as a function of interpreter use (interpreter vs no interpreter), age, gender (female vs male), race (Black, Asian, Hispanic/Latino, American Indian/Alaskan, Native/Native Hawaiian, other vs White), method of arrival to the ED (ambulance, public transportation/car, police, hospital transport, medical flight, other), insurance (Medicare, Medicaid, private insurance, uninsured), with an interaction between race and use of an interpreter. Adjusted odds ratios (aOR) and 95% confidence intervals (CI) are presented for all model covariates. A more parsimonious multivariable model was run with race and LEP status as regressors prior to adjusting for age, gender, insurance, status, and method of admission to the ED.

We assessed multicollinearity between race and being LEP by estimating variance inflation factors (VIF) to assess whether both variables should be included in the model, as it was thought that certain races recorded may be more likely to use an interpreter. A type I error of 5% was used for all CIs and hypothesis tests. We performed all statistical analysis in RStudio version 1.2.1335 (Boston, MA) and Prism version 9.3.0 (GraphPad Software, San Diego, CA).

## RESULTS

### Study Cohort

A total of 114,447 patients presented to the ED during the study period, of whom 85,899 were eligible for inclusion in the study (Figure, Table 1). A total 9,874 (11.5%) were LEP, and 76,025 (88.5%) were English proficient (EP). Of the eligible patients, 3,179 (3.70%) were admitted for surgery, of whom 373 (11.7%) were LEP. Mean age was significantly higher in the group admitted for surgery compared to the unadmitted group (48.8 vs 47.1, respectively; *P*<0.001; median age overall 44 interquartile ratio=36), although this distinction is unlikely to bear clinical significance. There were 41,299 (48.1%) self-reported females in the total sample, and 1,459 (45.9%) of those admitted for surgery were female (*P*=0.01) (Table 1).

Of the study population 9,949 (ll.6%) self-reported they were Black, and 54,307 (63.2%) reported they were White. Of the individuals admitted for surgery, 73.5% (2,338) were White, 5.92% (188) were Black, 4.38% (139) were Asian, and 4.85% (154) were Hispanic. Importantly, 4.31% (2,338/54,307) of individuals identifying as White were admitted for surgery, 1.89% (188/9,949) of individuals identifying as Black were admitted for surgery, 3.69% (154/4,168) of Hispanic individuals were admitted for surgery, and 3.19% (139/4,364) of individuals identifying as Asian were admitted for surgery. Of all individuals admitted for surgery, 1,304 (41.0%) arrived via public transit or car and 1,526 (48.0) arrived by Ambulance.

### Key Results

The simpler multivariable logistic regression model found that LEP individuals had significantly higher odds of admission for surgery compared to EP individuals (OR 1.33, CI 1.17–1.50; *P*<0.005) (Table 2). However, after adjusting for age, gender, method of arrival to the ED, race, and insurance status, the analysis failed to detect a significant difference in the number of individuals with LEP who were admitted for surgery from the ED compared to those with English proficiency (3.78% vs 3.69%; *P*=0.69). The odds of admission for surgery were significantly lower for patients who self-reported Black or Asian race (aOR, 0.456, CI 0.388–0.533, *P*<0.005, and aOR 0.759, CI 0.612–0.929; *P*=0.009, respectively). Females were significantly less likely to be admitted for surgery compared to males (aOR 0.926, CI 0.862, 0.996, *P*=0.04). Patients were more likely to be admitted for surgery if they had private insurance (aOR 1.25, CI 1.13–1.39; *P*<0.005) and less likely if they were uninsured (aOR 0.581, CI 0.323–0.958; *P*=0.05).

Patients were also less likely to be admitted for surgery if they arrived by public transportation or car (aOR 0.443, CI 0.409–0.479]; *P*<0.001) when compared to arrival by ambulance. Conversely, subjects were more likely to be admitted if they arrived via medical flight (aOR 7.88, CI 6.37–9.72; *P*<0.005). Interestingly, despite self-reported Hispanic race not being significant independently, a significant interaction was reported among LEP individuals who were Hispanic (aOR 1.63, CI 1.08–2.47; *P*=0.02), suggesting that Hispanic individuals who were also LEP were more likely to be admitted for surgery than their non-LEP counterparts. Variance inflation factors assessed in the dual-variable model revealed no significant multicollinearity (VIF_LEP_=1.06; VIF_race_=1.02).

## DISCUSSION

In this study of the association between race, LEP, and admission for surgery from the ED, multivariable logistic regression analysis determined that individuals self-identifying as Black or Asian had significantly lower odds of admission compared to individuals self-identifying as white. There was no evidence of a significant difference in the odds of admission for surgery among LEP compared to EP patients. We also found significantly lower odds of direct admission from the ED for surgery on individuals self-identifying as female and those without insurance, whereas individuals with private insurance had significantly higher odds of admission for surgery.

The fact that racial minorities experience lower rates of healthcare utilization^17^ and poorer postoperative outcomes^18^ is well characterized. However, to our knowledge, this is the first study finding that minorities are less likely to be admitted for emergent surgery from the ED, a time when indications for care are thought to be less dependent on subjective measures and judgments known to introduce bias (eg, pain ratings).^19^ Future studies should be performed to better understand what factors are driving lower admission rates for surgery among minorities and women, looking specifically at measures of discrimination among patients in the ED.

There are several reasons why racial and lingual minorities may have lower odds of admission for surgery. Disparities in rates of surgery between minorities and Whites have been previously reported in accountable care organizations.^17^ Such disparities may stem from systemic racism within health systems, differential levels of access to ED care, varying clinician assessments of minorities’ pain levels,^20^ or varying levels of health literacy among LEP communities.^21^ Another possible explanation is that racial minorities are less likely to have access to a primary care physician, which then leads them to use the ED as a first point of care. This has been shown in multiple studies and is known to influence admission rates to the hospital from the ED and ED presentation.^22^–^24^ Another explanation is that clinician biases lead to differences in the assessment and triage of patients who are at risk of needing emergent surgery, which could lead to either a decrease in the percentage of Black and Asian patients admitted for surgery from the ED overall or a delay in admission for surgery, which would not have been detected in this study because we limited admission for emergent surgery to one day after admission.

We found no evidence of a significant difference in admission for surgery in LEP individuals compared to EP individuals. In the context of non-emergent surgery, other researchers have found that LEP individuals are significantly less likely to pursue surgical treatment options.^25^ However, Ngai et al, who examined rates of inpatient admission from the ED in LEP and EP individuals, found no significant difference in admission rates between the two groups but did detect a significant increase in unplanned readmissions among the LEP group compared to the EP group.^9^ However, a recent systematic review suggests that any increase in readmissions among LEP individuals may be concentrated to the setting of chronic disease (eg, heart failure) but not for surgeries or acute procedures.^26^ Taken together, the paucity of existing data as well as the findings of this study suggest no difference in admission from the ED but disparities elsewhere in the care process. One hypothesis to explain this may be that indications for admission for surgery are not always dependent on communication between the patient and clinician; however, this was not measured in this study or the cited studies herein.

All females in this study were less likely to be admitted for surgery from the ED, despite making up 48.1% of the sample. This could be a result of documented discriminatory practices among women (in particular minority women).^27^ The results obtained for the odds of admission for surgery based on the patient’s method of arrival to the ED were expected, as it is more likely that an individual arriving via hospital transport or medical flight is in a more severe condition and in need of surgery than one arriving via public transportation or car.

Uninsured individuals were significantly less likely to be admitted for emergent surgery, while those with private insurance were significantly more likely to be admitted for emergent surgery in this study. Despite common perceptions to the contrary, research suggests that uninsured individuals use the ED at comparable rates to their insured counterparts^28^ but do receive other forms of care less frequently than those with insurance,^29^ suggesting that overall utilization rates alone are unlikely to explain the admission rates for surgery found. Although substantial data exists suggesting that the uninsured experience worse outcomes after surgery,^30^ little data exists that sheds light on why uninsured individuals may have lower odds of admission for emergent procedures. It is possible that this is a decision rooted in implicit considerations of lower reimbursements and worse expected outcomes, but it is also possible that patients without insurance choose not to go to the ED in the first place because of the costs associated with receiving care without insurance.

The fact that Hispanic individuals who were also LEP were more likely to be admitted for surgery than their non-LEP counterparts deserves further exploration. Similarly, the fact that those identifying as Black or Asian had significantly lower odds of admission for emergent surgery while those identifying as Hispanic did not should also be re-examined in future studies. We believe the most likely explanation for this variation is found in the small sample sizes of our racial minority groups compared to the group identifying as White. However, this result should be replicated in future studies to assess its generalizability across institutions.

## LIMITATIONS

This study has several limitations. First, the data is from a single institution and is retrospective in design, limiting its generalizability to other institutions and preventing it from making any causal conclusions. Second, this data did not account for individuals who may have been LEP themselves but arrived with a family member or friend who could translate for them, which would preclude these individuals from being identified as LEP in this study. This also includes scenarios in which residents or attending physicians may have spoken the patient’s language fluently and opted not to use an interpreter. Further, approximately 10% of the initial pre-filtered study sample was excluded because those patients did not report whether or not they required an interpreter, and 3.9% of individuals in the initial pre-filtered study sample had unavailable or missing race data. We do not believe these omissions had significant effects on the results of this study, as the percentage of missing race data is minimal. Further, it likely that most individuals who did not report requiring an interpreter did not require interpreter services (as it is mandated by law to provide an interpreter, which would be reported in the chart). These factors would likely influence the number of patients counted as LEP and could thus skew the results obtained. However, all individuals who reported that English was not their primary language used an interpreter; thus, we believe the potential effects of excluding this group are minor.

We did not assess why minorities, women, and the uninsured were less likely to be admitted for emergent surgery from the ED, which now represents a major area of research for future studies. Further, our analysis includes primarily socioeconomic variables, and it is important to consider for future studies that there may be myriad other clinical factors that influence admission that were not reported here. Another limitation of this study is that the patients were not stratified based on their admitting chief complaint or time of admission throughout the week. It is possible that there may be a difference in emergent surgery admission rates in institutions that practice surgical smoothing (eg, delaying some cases, such as cholelithiasis, to be performed on Monday instead of immediately over the weekend) vs those that do not. Future studies should take the opportunity to compare the most prevalent chief complaints in the ED to see whether the results herein hold for patients presenting with similar problems.

This study demonstrates that disparities in rates of admission for emergent surgery from the ED exist and may be a contributive variable in existing health disparities within ED care. The differences documented may reflect larger differences in rates of presentation to the ED among racial and ethnic minorities, and it serves as one potential explanation for why many racial and ethnic minorities are hesitant to receive care in the ED. Regardless, this study highlights the need for both further study and institutional reflection on practices of evaluation and admission for emergent procedures from the ED.

## CONCLUSION

We found that individuals identifying as being female, Black, Asian, or uninsured have significantly lower odds of direct admission for surgery from the ED. We did not find evidence that individuals with limited English proficiency status were more or less likely to be admitted for emergent surgery compared to their EP counterparts. Further studies are needed to clarify what other factors influence a patient’s admission for surgery outside of race, gender, and insurance status. Further studies are also needed to elicit the causal factors for admission for surgery from the ED.

## Figures and Tables

**Figure:**
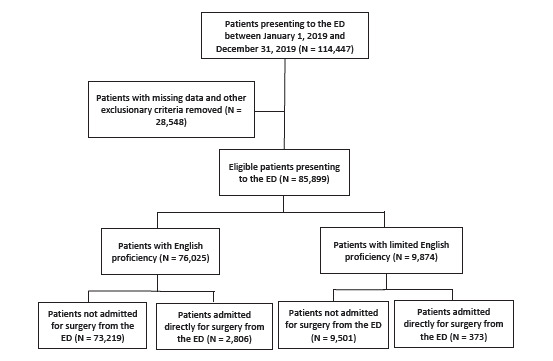
Study cohort flow chart for patients admitted from the emergency department (ED) to surgery.

**Table 1:** Descriptive statistics stratified by admission for surgery from the ED.

	Admitted for surgery (N = 3,179)	Non-surgical patients (N = 82,720)	Combined (N = 85,899)	Percentage of patients in each sub-category admitted for surgery (%)	*P*-value
Age (SD)	49.36 (23.67)	45.16 (23.06)	45.31 (23.09)		<0.001
Gender					0.01
Male	1,720 (54.1)	42,880 (51.8)	44,600 (51.9)	3.86	
Female	1,459 (45.9)	39,840 (48.2)	41,299 (48.1)	3.53	
Race					<0.001
White	2,338 (73.5)	51,969 (62.8)	54,307 (63.2)	4.31	
Black	188 (5.9)	9,761 (11.8)	9,949 (11.6)	1.89	
Asian	139 (4.4)	4,225 (5.1)	4,364 (5.1)	3.19	
Hispanic	154 (4.8)	4,014 (4.9)	4,168 (4.9)	3.69	
American Indian/Alaska Native/Native Hawaiian	7 (0.2)	384 (0.5)	391 (0.5)	1.79	
Other	353 (11.1)	12,367 (15.0)	12,720 (14.8)	2.78	
Method of ED arrival					<0.001
Ambulance	1,526 (48.0)	24,550 (29.7)	26,076 (30.4)	5.85	
Public transport/car	1,304 (41.0)	48,238 (58.3)	49,542 (57.7)	2.63	
Police	0 (0.0)	130 (0.2)	130 (0.2)	0.0	
Medical flight	141 (4.4)	271 (0.3)	412 (0.5)	34.2	
Hospital transport	27 (0.8)	358 (0.4)	385 (0.4)	7.01	
Other	181 (5.7)	9,173 (11.1)	9,354 (10.9)	1.94	
Insurance status					<0.001
Medicare	1,014 (31.9)	22,532 (27.2)	23,546 (27.4)	4.31	
Medicaid	468 (14.7)	16,966 (20.5)	17,434 (20.3)	2.68	
Private insurance	1,683 (52.9)	42,397 (51.3)	44,080 (51.3)	3.82	
Uninsured	14 (0.4)	825 (1.0)	839 (1.0)	1.67	
Interpreter use					0.69
Yes	373 (11.7)	9,501 (11.5)	9,874 (11.5)	3.78	
No	2,806 (88.3)	73,219 (88.5)	76,025 (88.5)	3.69	

Note: Differences in the total number of individuals in a category are due to different numbers of patients who were excluded from the final sample after study criteria were applied to the total sample. P-values represent comparisons of the distributions for each category, not pairwise comparisons of each subcategory. Values represent the total number of individuals (percentage). Number of participants that were excluded due to missing data for each variable: age (0); gender (0); race (4,087); method of ED arrival (51); insurance status (0); interpreter use (0). *ED*, emergency department; *SD*, standard deviation.

**Table 2:** Overall multivariable logistic regression results.

Variable	Simple model		Fully adjusted model	
	OR (95% CI)	P-value	aOR (95% CI)	*P*-value
Intercept	0.0446 (0.0427, 0.0464)	< 0.005	0.0629 (00516, 0.0766)	< 0.005
Limited English proficiency				
(Interpreter required)	1.33 (1.17, 1.50)	< 0.005	0.994 (0.769, 1.264)	0.96
Age	--	--	1.00 (1.00,1.00)	0.02
Gender (Female)	--	--	0.926 (0.862, 0.996)	0.04
Arrival method	--	--		
Ambulance			Reference	Reference
Public transportation/car			0.443 (0.409, 0.479)	< 0.005
Police			0.00000839 (0.00, 0.000119)	0.88
Hospital transport			1.23 (0.807, 1.79)	0.31
Medical flight			7.88 (6.37, 9.72)	< 0.005
Other			0.321 (0.273, 0.374)	< 0.005
Race				
White	Reference	Reference	Reference	Reference
Black	0.422 (0.362, 0.489)	< 0.005	0.456 (0.388, 0.533)	< 0.005
Asian	0.689 (0.575, 0.818)	< 0.005	0.759 (0.612, 0.929)	0.009
Hispanic/Latino	0.787 (0.661, 0.930)	0.00591	0.828 (0.664, 1.02)	0.09
American Indian/Alaska Native/Native Hawaiian	0.404 (0.173, 0.789)	0.0177	0.499 (0.213, 0.979)	0.07
Other	0.564 (0.496, 0.639)	< 0.005	0.610 (0.517, 0.715)	< 0.005
Insurance status	--	--		
Medicare			Reference	Reference
Medicaid			0.877 (0.766, 1.002)	0.06
Private insurance			1.25 (1.13, 1.39)	< 0.005
Uninsured			0.581 (0.323, 0.958)	0.05
LEP: Race interaction	--	--		
LEP:White			Reference	Reference
LEP:Black			1.29 (0.726, 2.20)	0.36
LEP:Asian			1.36 (0.862, 2.12)	0.18
LEP:Hispanic/Latino			1.63 (1.08, 2.47)	0.02
LEP:American Indian/Alaska Native/Native Hawaiian			1.90 (NA, NA)	0.96
LEP:Other			1.51 (1.10, 2.11)	0.01

*CI*, confidence interval; *OR*, odds ratio; *aOR*, adjusted odds ratio; *LEP*, limited English proficiency.

## References

[1] Yearby R (2018). Racial disparities in health status and access to healthcare: the continuation of inequality in the United States due to structural racism. Am J Econ Sociol.

[2] Wang TF, Shi L, Nie X (2013). Race/ethnicity, insurance, income and access to care: the influence of health status. Int J Equity Health.

[3] Staton LJ, Panda M, Chen I (2007). When race matters: disagreement in pain perception between patients and their physicians in primary care. J Natl Med Assoc.

[4] Joynt KE, Orav EJ, Jha AK (2011). Thirty-day readmission rates for Medicare beneficiaries by race and site of care. JAMA.

[5] Williams DR, Lawrence JA, Davis BA (2019). Racism and health: evidence and needed research. Annu Rev Public Health.

[6] McHugh P, Zeigler K, Camarota S (2019). 67.3 Million in the United States spoke a foreign language at home in 2018.

[7] Fox MT, Godage SK, Kim JM (2020). Moving from knowledge to action: improving safety and quality of care for patients with limited English proficiency. Clin Pediatr (Phila).

[8] Tang EW, Go J, Kwok A (2016). The relationship between language proficiency and surgical length of stay following cardiac bypass surgery. Eur J Cardiovasc Nurs.

[9] Ngai KM, Grudzen CR, Lee R (2016). The association between limited English proficiency and unplanned emergency department revisit within 72 hours. Ann Emerg Med.

[10] Juergens CP, Dabin B, French JK (2016). English as a second language and outcomes of patients presenting with acute coronary syndromes: results from the CONCORDANCE registry. Med J Aust.

[11] Massachusetts General Hospital About the Emergency Department.

[12] Adib CT (2019). Characterization, prediction, and mitigation of code help events at Massachusetts General Hospital.

[13] Kobayashi KJ, Knuesel SJ, White BA (2019). Impact on length of stay of a hospital medicine emergency department boarder service. J Hosp Med.

[14] Epic Systems Epic Systems.

[15] Worster A, Bledsoe RD, Cleve P (2005). Reassessing the methods of medical record review studies in emergency medicine research. Ann Emerg Med.

[16] Gilbert EH, Lowenstein SR, Koziol-McLain J (1996). Chart reviews in emergency medicine research: Where are the methods?. Ann Emerg Med.

[17] Schoenfeld AJ, Sturgeon DJ, Dimick JB (2019). Disparities in rates of surgical intervention among racial and ethnic minorities in Medicare accountable care organizations. Ann Surg.

[18] Esnaola NF, Hall BL, Hosokawa PW (2008). Race and surgical outcomes: It Is not all black and white. Ann Surg.

[19] Lee P, Le Saux M, Siegel R (2019). Racial and ethnic disparities in the management of acute pain in US emergency departments: Meta-analysis and systematic review. Am J Emerg Med.

[20] Cintron A, Morrison RS (2006). Pain and ethnicity in the United States: a systematic review. J Palliat Med.

[21] Sentell T, Braun KL (2012). Low health literacy, limited English proficiency, and health status in Asians, Latinos, and other racial/ethnic groups in California. J Health Commun.

[22] Zhang X, Carabello M, Hill T (2020). Trends of racial/ethnic differences in emergency department care outcomes among adults in the United States from 2005 to 2016.

[23] Institute of Medicine (US) Committee on Understanding and Eliminating Racial and Ethnic Disparities in Health Care (2003). Unequal Treatment: Confronting Racial and Ethnic Disparities in Health Care.

[24] Heron SL, Stettner E, Haley LL (2006). Racial and ethnic disparities in the emergency department: a public health perspective. Emerg Med Clin North Am.

[25] Betjemann JP, Thompson AC, Santos-Sánchez C (2013). Distinguishing language and race disparities in epilepsy surgery. Epilepsy Behav.

[26] Woods AP, Alonso A, Duraiswamy S (2022). Limited English proficiency and clinical outcomes after hospital-based care in English-speaking countries: a systematic review. J Gen Intern Med.

[27] Marshall KJ, Urrutia-Rojas X, Mas FS (2005). Health status and access to health care of documented and undocumented immigrant Latino women. Health Care Women Int.

[28] Newton MF, Keirns CC, Cunningham R (2008). Uninsured adults presenting to US emergency departments: assumptions vs data. JAMA.

[29] Zhou RA, Baicker K, Taubman S (2017). the uninsured do not use the emergency department more—they use other care less. Health Aff (Millwood).

[30] Schwartz DA, Hui X, Schneider EB (2014). Worse outcomes among uninsured general surgery patients: Does the need for an emergency operation explain these disparities?. Surgery.

